# Efficacy of Recombinant Human Parathyroid Hormone versus Vertebral Augmentation Procedure on Patients with Acute Osteoporotic Vertebral Compression Fracture

**DOI:** 10.1111/os.13470

**Published:** 2022-08-26

**Authors:** Pengguo Gou, Zhihui Zhao, Chen Yu, Xuefeng Hou, Gang Gao, Ting Zhang, Feng Chang

**Affiliations:** ^1^ Department of Orthopedic Surgery The Fifth Affiliated Hospital of Shanxi Medical University Taiyuan Shanxi China; ^2^ Department of Orthopedic Surgery The Tianjin 4th Centre Hospital Tianjin Tianjin China

**Keywords:** fracture healing, osteoporosis, parathyroid hormone, procedures, spine

## Abstract

**Objective:**

Although widely used in clinical practice, vertebral augmentation procedure (VAP) for osteoporotic vertebral compression fracture (OVCF) is not supported. Recently, the effect of recombinant human parathyroid hormone (1–34) (rhPTH) has been paid great attention for its efficacy in anti‐osteoporosis and bone union. This study aims to explore the outcome of rhPTH on acute OVCF and compare it with VAP to clarify its therapeutic advantages.

**Methods:**

The retrospective study comprised 71 acute OVCF patients from January 2015 to March 2020: 22 received rhPTH treatment (rhPTH group) and 49 underwent VAP (VAP group). The rhPTH group was 15 women and seven men with an average of 76.18 years, and the VAP group were 35 women and 14 men with an average of 73.63 years. The thoracic/lumbar vertebrae were 14/8 in the rhPTH group and 29/20 in the VAP group. The average follow‐up period was 14.05 months in the rhPTH group and 13.82 months in the VAP group. The two groups were assessed regarding the visual analog score (VAS), Oswestry Disability Index (ODI), OVCF bone union, bone mineral density (BMD), kyphotic angle (KA), anterior and posterior border height (ABH and PBH, respectively), adverse events and the health‐related quality of life assessed by short form‐36 health survey scores (SF‐36). Categorical variables were analyzed by chi‐square test and continuous variables between groups were analyzed by independent samples *t*‐test or Mann–Whitney *U* test according to the normality.

**Results:**

During the follow‐up, the VAS was significantly lower in the rhPTH group than in the VAP group at month 3 (0.39 ± 0.6 *vs* 0.68 ± 0.651) (*p* = 0.047), month 6 (0.45 ± 0.60 *vs* 2.18 ± 1.22) (*p* < 0.001), and month 12 (0.45 ± 0.60 *vs* 2.43 ± 1.49) (*p* < 0.001). At month 12, the ODI was significantly lower in the rhPTH group (18.59 ± 3.33%) than in the VAP group (28.93 ± 16.71%) (*p* < 0.001). Bone bridge was detected on sagittal computed tomography images of all fractured vertebrae in the rhPTH group. The BMD was significantly higher in the rhPTH group (87.66 ± 5.91 Hounsfield units [HU]) than in the VAP group (68.15 ± 11.32HU) (*p* < 0.001). There were no significant differences in the changes in KA, ABH, and PBH between groups (all *p* > 0.05). The incidence of new OVCF was significantly lower in the rhPTH group than in the VAP group (*p* = 0.042). All scores of SF‐36 were significantly higher in the rhPTH group than in the VAP group (all *p* < 0.05).

**Conclusion:**

In acute OVCF patients, rhPTH was better than VAP in increasing spinal BMD to promote OVCF healing, reduce new OVCF, and improve back pain, physical ability, and health‐related quality of life.

## Graphical abstract

Bone union assessed by ImageJ after rhPTH treatment. The original sagittal CT image of L2 (A, arrowhead). The region of interest is drawn according to the L2 outline (B, yellow line). Bone bridge connecting the upper and lower endplates is detected by setting the CT values of 200–1000 HU (C, red color region), indicating bone healing.

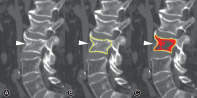

## Introduction

Osteoporotic vertebral compression fracture (OVCF) is the most common fragility fracture, accounting for almost 50% of all osteoporotic fractures^1^. The estimated incidence in individuals >50 years of age was 307/100,000 per year based on a German study^2^. Symptomatic OVCF patients commonly suffer from significant and long‐lasting back pain, substantially impacting patients' health‐related quality of life (HRQoL)^3^, ^4^, ^5^, ^6^. In addition to increased morbidity and mortality, OVCF imposes a significant economic burden on the public health systems worldwide and patient families^7^, ^8^, ^9^.

Traditional conservative treatment, including pain medication, bed rest, and bracing, is an option for symptomatic OVCF^10^. However, approximately 30%–40% of patients still experience severe back pain following the healing of OVCF^11^, ^12^. The progressive vertebral collapse was not rare after conservative treatment. In addition, not all OVCF healed following this treatment. Once nonunion occurs, patients may suffer from intractable back pain and neurological deficits, leading to a further reduced HRQoL and increased mortality^13^, ^14^.

Vertebral augmentation procedure (VAP) was widely applied to stabilize the fractured vertebrae to relieve back pain immediately, considering the drawbacks of conservative treatment^15^. However, recent high‐ to moderate‐quality evidence indicates that VAP does not offer significant clinical benefits compared with the sham procedure^16^, ^17^. One challenge was that VAP treatment only targeted the restoration of stability of the fractured vertebrae and did not improve vertebral bone mineral density (BMD) to decrease the fracture risk of the treated or non‐treated vertebrae^18^. Another challenge is that VAP is not suitable for patients with surgical contraindications^19^. In addition, the prevalence of severe complications related to VAP could be as high as 12.5%–36.8%^20^, ^21^, ^22^, ^23^, ^24^.

Recombinant human parathyroid hormone (1–34) (rhPTH), the only anabolic drug in all anti‐osteoporosis agents, has been widely employed for osteoporosis with a high fracture risk^25^. Daily injection of rhPTH can stimulate intravertebral bone formation to increase BMD and subsequent bone strength, thereby reducing the fracture risk of the vertebral body^26^, ^27^. Recently, the effects of short‐term rhPTH treatment on the vertebral collapse in acute OVCF nonunion patients have been reported^28^.

A better choice for OVCF is not only to alleviate clinical symptoms by restoring vertebral stability but also to improve the BMD to decrease the risk of vertebral fracture, especially in patients with surgical contraindications. In theory, rhPTH treatment could achieve a good therapeutic effect by promoting the new bone formation of fractured vertebrae to boost fracture union and improve the spinal BMD to decrease fracture risk. However, the evidence to evaluate the therapeutic effect following rhPTH treatment in patients with acute OVCF was limited.

Therefore, our study aimed to: (i) explore the outcome of rhPTH on acute OVCF as to the back pain, physical ability, fracture union, changes in BMD, kyphotic angle (KA), vertebral height, adverse events and HRQoL; and (ii) compare with VAP to illustrate the treatment advantages of rhPTH.

## Materials and Methods

### Patients

This study retrospectively reviewed the medical records of acute OVCF patients receiving rhPTH or VAP treatment between January 2015 and March 2020 to compare the clinical efficacy. The study was approved by the Institutional Review Board of the Fifth Affiliated Hospital of Shanxi Medical University (approval No. 2022–199).

### Inclusion and Exclusion Criteria

Inclusion criteria: (i) age 60–90 years; (ii) acute OVCF (<2 weeks^29^) from low energy trauma (fall from a standing height or less^30^, ^31^); (iii) patients suffering from severe back pain; (iv) only underwent either rhPTH or VAP treatment.

Exclusion criteria: (i) history of taking drugs affecting bone metabolism; (ii) vertebral infections or tumor; (iii) history of spinal surgery; (iv) OVCF with spinal cord dysfunction; (v) endplate Modic changes.

Finally, 22 patients who received rhPTH treatment (rhPTH group) and 49 patients who underwent VAP (VAP group) were included in this study. All the patients' data were collected and measured by two experienced orthopaedic surgeons (T. Z. and G. G). Both readers were blinded to all the clinical and imaging data.

### Radiological Diagnosis

Magnetic resonance imaging (MRI) was performed and used for OVCF diagnosis, on which the bone marrow signals displayed hypointensity on T1‐weighted and hyperintensity on T2‐weighted and fat‐suppressed sequences^32^, ^33^, ^34^. Given that T10‐L2 vertebra levels are the most common fracture site, CT values of L4 ≤ 80 Hounsfield unit (HU) were applied to the osteoporosis diagnosis^35^.

### Treatment Method

Patients in the rhPTH group received once daily rhPTH administered by 20μg subcutaneous injection in the morning for at least 6 months in the rhPTH group. Commonly, patients received rhPTH treatment within their community, not requiring hospitalization. Patients in the VAP group were administered VAP treatment in the VAP group. No patients in both groups were administered analgesics following the treatment. Calcium (1.2g/day) and vitamin D (800 IU/day) were administrated in both groups.

### Clinical Outcomes Assessment

The visual analog score (VAS) was used to evaluate each patient's back pain at baseline, month 1, 3, 6, and 12 following treatment^36^. The Oswestry Disability Index (ODI) was employed to assess each patient's physical ability at baseline and month 12^37^.

### Fracture Union Assessment

**Fig. 1:**
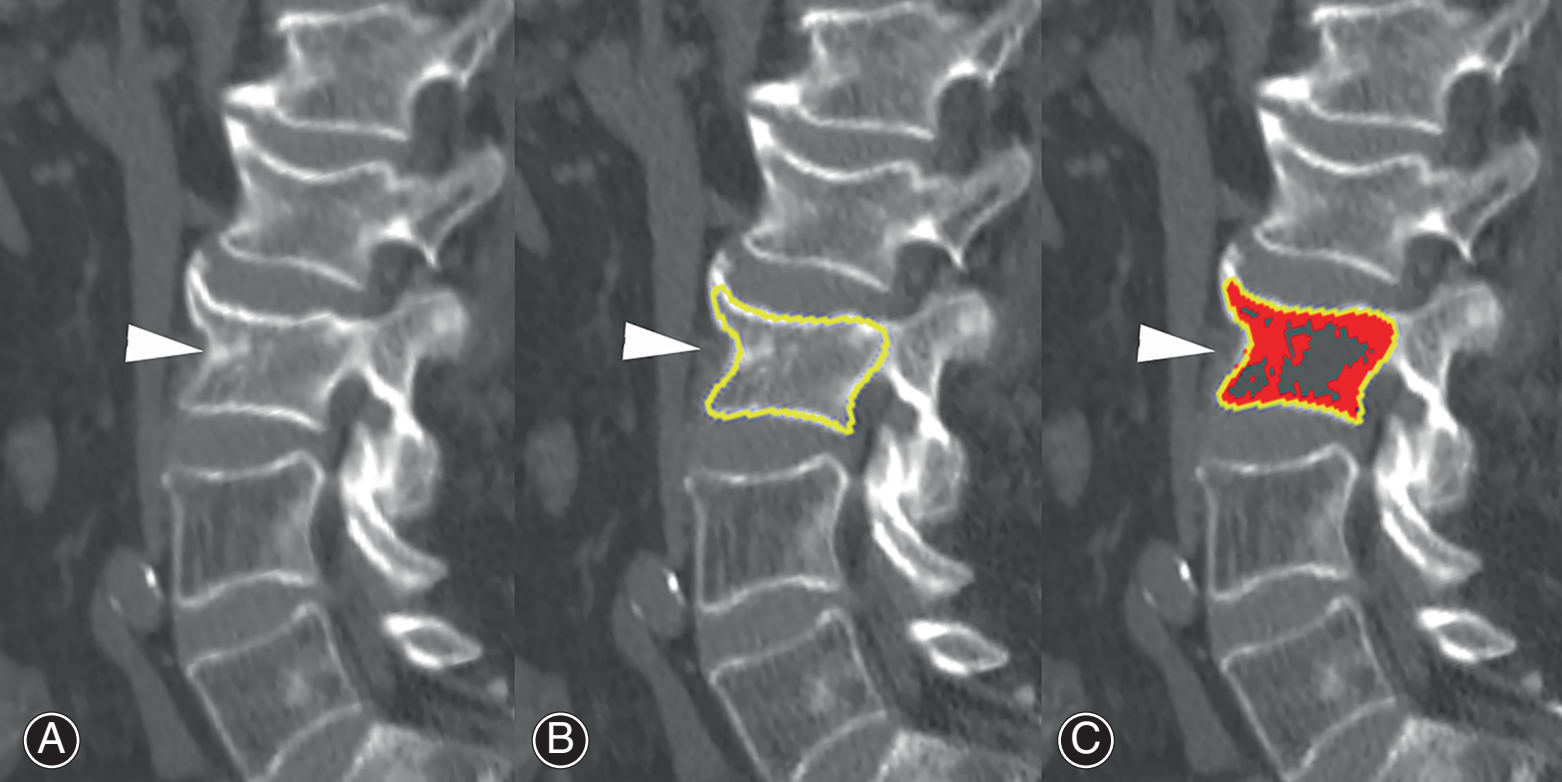
Fracture union assessed by ImageJ after rhPTH treatment. The original sagittal CT image of L2 (A, arrowhead). The region of interest is drawn according to the L2 outline (B, yellow line). Bone bridge connecting the upper and lower endplates is detected by setting the CT values of 200–1000 HU (C, red color region), indicating bone healing. HU, Hounsfield unit

Bone healing of OVCF was defined as the recovery of bone continuity detected on sagittal CT sections at month 4 following treatment, in which the formation of a bone bridge connected the upper and lower endplates. Sagittal CT images of the fractured vertebrae were quantitatively assessed with ImageJ (https://imagej.nih.gov/ij/) with a HU ratio of 200–1000 to evaluate bone bridge after rhPTH treatment^28^, ^38^. The region of interest (ROI) was drawn according to the vertebral outline after importing the image. Bone tissue within the ROI was detected by setting the CT values of 200–1000 HU and was marked in red. The red region connecting the upper and lower endplates indicated the bone bridge (Fig. 1). Bone union assessment of the cemented vertebrae was not performed in the VAP group because the assessment of the bone bridge was affected by high‐density bone cement.

### KA and Vertebral Height Assessment

The KA, anterior and posterior border height (ABH and PBH) of the fractured vertebrae were analyzed on the lateral X‐rays. The KA of the fractured vertebrae was measured using the angle of the endplate line. The loss rate of vertebral height and the increment in KA were reviewed at baseline and month 12, respectively. The loss rate of vertebral height (%) = [(vertebral height)_baseline_ − (vertebral height)_month 12_ × 2] × 100/[(upper vertebral height + lower vertebral height)].

### BMD Evaluation

The CT value of L4 quantified on sagittal CT images was used to evaluate the spinal BMD at baseline and month 12^35^.

### Adverse Events Evaluation

During follow‐up, the adverse events were recorded, including new OVCF and such serious adverse events affecting the continuation of treatment as severe gastrointestinal disorders, major adverse cardiac events, and severe dizziness.

### HRQoL Assessment

The Short Form‐36 Health Survey (SF‐36) was employed for evaluating each patient's HRQoL at baseline and month 12^39^. The SF‐36 is composed of the physical component summary (PCS) scores (physical functioning [PF], role physical [RP], bodily pain [BP], and general health [GH]) and mental component summary (MCS) scores (social functioning [SF], mental health [MH], role physical [RE], and vitality [VT]).

### Power Calculation

Based on our pilot experiment, we assumed a normal distribution and VAS standard deviation (SD) of 1.50. With a two‐sided *α* = 0.05, a minimum sample size of 11 patients in each group gave a power of 0.9 to detect a mean difference of 1.50.

### Statistical Analyses

Data analysis were performed by SPSS version 21 (SPSS Inc.). Results were presented as mean ± SD. Interclass correlation coefficients (ICC) were analyzed to assess the extents of agreement of quantitative variables collected from the two readers. The average values of quantitative variables from the two readers were used for further analyses. Categorical variables were analyzed using the chi‐square tests. Independent samples *T*‐test or Mann‐Whitney *U* test were performed according to data normality. A paired *t*‐test and Wilcoxon signed‐rank test were performed to compare intragroup differences according to the data normality. Statistical significance was set at *p* < 0.05.

## Result

### Demographic Data

The demographic data between groups, including gender, age, the interval from injury to MRI study, location of OVCF, the period from OVCF to initial treatment, and follow‐up period, indicated no statistical differences (all *p* > 0.05). (Table 1).

**TABLE 1:** Patients' demographic data

	rhPTH group (*n* = 22)	VAP group (*n* = 49)	Test statistic	*p*‐value
Gender (female/male)	15/7	35/14	0.077	0.783
Age (years)	76.18 ± 5.43	73.63 ± 5.78	0.583	0.085
Interval from injury to MRI study (days)	4.55 ± 4.04	5.10 ± 4.01	0.080	0.591
Location of OVCF (thoracic/lumbar)	14/8	29/20	0.556	0.727
Period from OVCF to initial treatment (days)	6.59 ± 4.22	7.94 ± 4.23	0.039	0.218
Follow‐up period (months)	14.05 ± 1.13	13.82 ± 1.20	0.394	0.452

### Repeatability of Quantitative Variables

The inter‐observer agreements for quantitative variables between reader 1 and reader 2 were excellent. Table 2 shows all the results of inter‐observer agreements for all quantitative variables both in the rhPTH and VAP groups (all *p* < 0.001).

**TABLE 2:** Repeatability and intra‐observer agreement of quantitative variables

	rhPTH group			VAP group		
	ICC	95% CI	*p*‐value	ICC	95% CI	*p*‐value
VAS						
Baseline	0.782	0.320–0.836	<0.001	0.859	0.599–0.851	<0.001
Month 1	0.947	0.766–0.955	<0.001	0.893	0.679–0.885	<0.001
Month 3	0.892	0.59‐ 0.916	<0.001	0.913	0.736–0.908	<0.001
Month 6	0.922	0.674–0.934	<0.001	0.961	0.872–0.957	<0.001
Month 12	0.888	0.579–0.910	<0.001	0.898	0.691–0.890	<0.001
ODI						
Baseline	0.897	0.603–0.916	<0.001	0.856	0.598–0.852	<0.001
Month 12	0.809	0.380–0.856	<0.001	0.965	0.884–0.961	<0.001
KA						
Baseline	0.923	0.697–0.941	<0.001	0.867	0.624–0.863	<0.001
Month 12	0.871	0.424–0.885	<0.001	0.865	0.617–0.859	<0.001
ABH						
Baseline	0.898	0.616–0.922	<0.001	0.931	0.784–0.926	<0.001
Month 12	0.916	0.673–0.935	<0.001	0.896	0.689–0.889	<0.001
PBH						
Baseline	0.866	0.524–0.898	<0.001	0.847	0.554–0.834	<0.001
Month 12	0.812	0.387–0.860	<0.001	0.823	0.526–0.820	<0.001
BMD						
Baseline	0.962	0.839–0.970	<0.001	0.897	0.688–0.888	<0.001
Month 12	0.815	0.393–0.860	<0.001	0.823	0.515–0.814	<0.001
MCS						
Baseline	0.910	0.651–0.929	<0.001	0.811	0.481–0.800	<0.001
Month 12	0.804	0.353–0.843	<0.001	0.903	0.697–0.893	<0.001
PCS						
Baseline	0.966	0.850–0.972	<0.001	0.925	0.767–0.919	<0.001
Month 12	0.899	0.591–0.915	<0.001	0.894	0.686–0.888	<0.001

Abbreviations: ABH, anterior border height; BMD, bone mineral density; ICC, interclass correlation coefficients; CI, confidence intervals; KA, kyphotic angle; MCS, mental component summary; ODI, Oswestry Disability Index; PBH, posterior border height; PCS, physical component summary; VAS, visual analog score.

### Clinical Outcomes

**Fig. 2:**
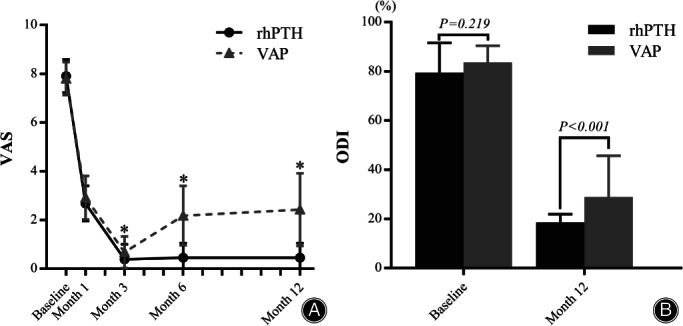
Changes in VAS (A) and ODI (B) before and after treatment between groups at different time points, respectively. *Means difference between groups was significant (*p* < 0.001)

No significant differences in VAS were found between the rhPTH and VAP groups at baseline (7.91 ± 0.68 *vs* 7.80 ± 0.68) (*p* = 0.568) and month 1 (2.68 ± 0.72 *vs* 2.92 ± 0.89) (*p* = 0.332). However, the VAS was significantly lower in the rhPTH group than the VAP group at month 3 (0.39 ± 0.61 *vs* 0.68 ± 0.65) (*p* = 0.047), month 6 (0.45 ± 0.60 *vs* 2.18 ± 1.22) (*p* < 0.001), and month 12 (0.45 ± 0.60 *vs* 2.43 ± 1.49) (*p* < 0.001) (Fig. 2).

No significant differences in ODI were found between the rhPTH (79.49 ± 12.05%) and the VAP group (83.67 ± 6.75%) at baseline (*p* = 0.219). At month 12, however, the ODI in the rhPTH group (18.59 ± 3.33%) was significantly lower than the VAP group (28.93 ± 16.71%) (*p* < 0.001) (Fig. 2).

From baseline to month 12, the decrement in VAS was significantly higher in the rhPTH group (7.45 ± 0.86) than in the VAP group (5.33 ± 1.74) (*p* < 0.001). The decrement in ODI was significantly higher in the rhPTH group (60.91 ± 11.67) than in the VAP group (54.74 ± 17.47) (*p* = 0.026).

### Fracture Union of OVCF

Sagittal CT images analyzed by ImageJ confirmed the radiographic fracture union in all affected vertebrae at month 4 following rhPTH treatment.

### KA, ABH, and PBH

**Fig. 3:**
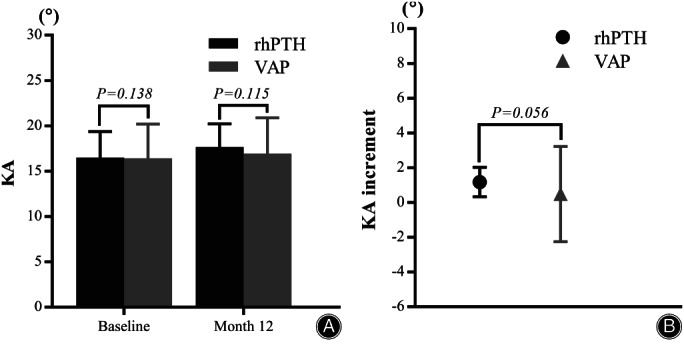
(A) No significant differences in KA were found between the rhPTH and the VAP groups at baseline or month 12 (both *p* > 0.05). (B) No significant differences in KA increment were found between groups from baseline to month 12 (*p* > 0.05). KA, kyphosis angle

Figure 3A showed no differences in KA between rhPTH and VAP groups (16.50 ± 2.87° *vs* 16.43 ± 3.76°) from baseline (*p* = 0.138) to month 12 (17.68 ± 2.55° *vs* 16.96 ± 3.93°) (*p* = 0.115). From baseline to month 12, no differences were observed in the KA increment in the rhPTH (1.18 ± 0.85°) and VAP groups (0.49 ± 2.74°) (*p* = 0.056) (Fig. 3B).

**Fig. 4:**
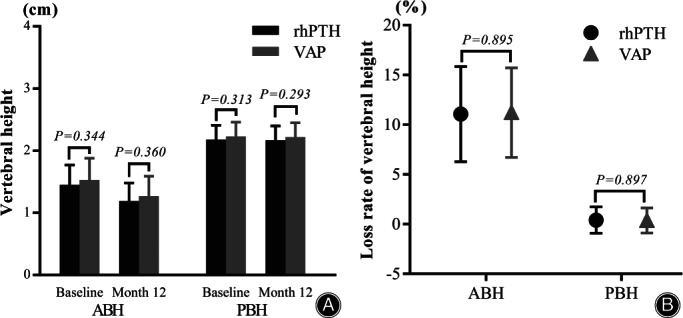
(A) No significant differences in the ABH and PBH were found between groups at baseline or month 12. (B) There were no significant differences in the loss rates of ABH and PBH between groups from baseline to month 12, respectively. ABH, anterior border height; PBH, posterior border height

There were no significant differences in ABH between the rhPTH and VAP groups at baseline (1.45 ± 0.32 *vs* 1.53 ± 0.35) (*p* = 0.344) and month 12 (1.19 ± 0.29 *vs* 1.27 ± 0.32) (*p* = 0.360), nor in PBH between groups at baseline (2.18 ± 0.23 *vs* 2.23 ± 0.23) (*p* = 0.313) and month 12 (2.17 ± 0.23 *vs* 2.22 ± 0.23) (*p* = 0.293) (Fig. 4A).

**Fig. 5:**
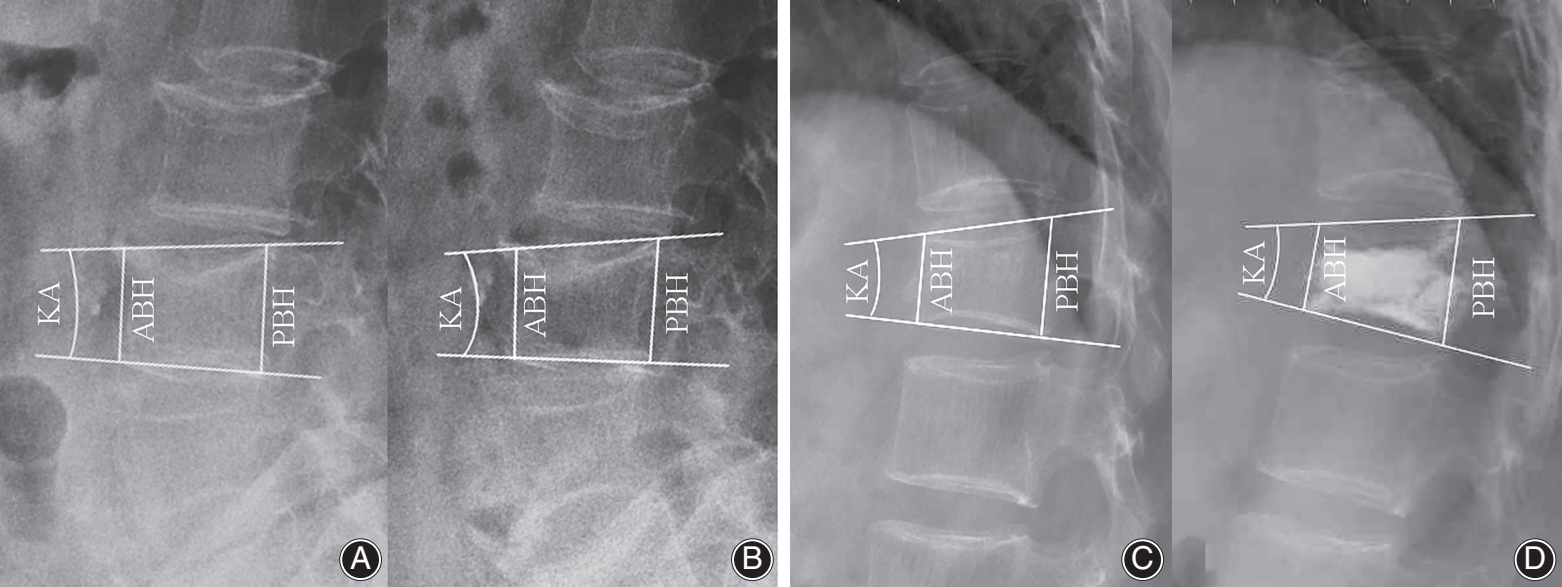
(A, B) The KA, ABH, and PBH in a 65‐year‐old female with acute L2 OVCF changes from 7°, 2.40, and 2.6 cm before treatment (A) to 9°, 2.25, and 2.55 cm following rhPTH at month 12 (B). (C, D) The KA, ABH, and PBH in a 67‐year‐old female with fresh T12 OVCF changes from 12°, 2.00, and 2.50 cm before treatment (C) to 13°, 1.85, and 2.50 cm after VAP treatment at month 12 (D). ABH, anterior border height; KA, kyphosis angle; PBH, posterior border height

From baseline to month 12, no significant differences in the loss rate of ABH were found between the rhPTH (11.05 ± 4.78%) and VAP groups (11.20 ± 4.50%) (*p* = 0.895), nor the loss rate of PBH between the rhPTH (0.41 ± 1.33%) and VAP group (0.37 ± 1.25%) (*p* = 0.897) (Fig. 4B). Figure 5 shows typical cases of changes in KA, ABH, and PBH following rhPTH and VAP treatments.

### BMD

**Fig. 6:**
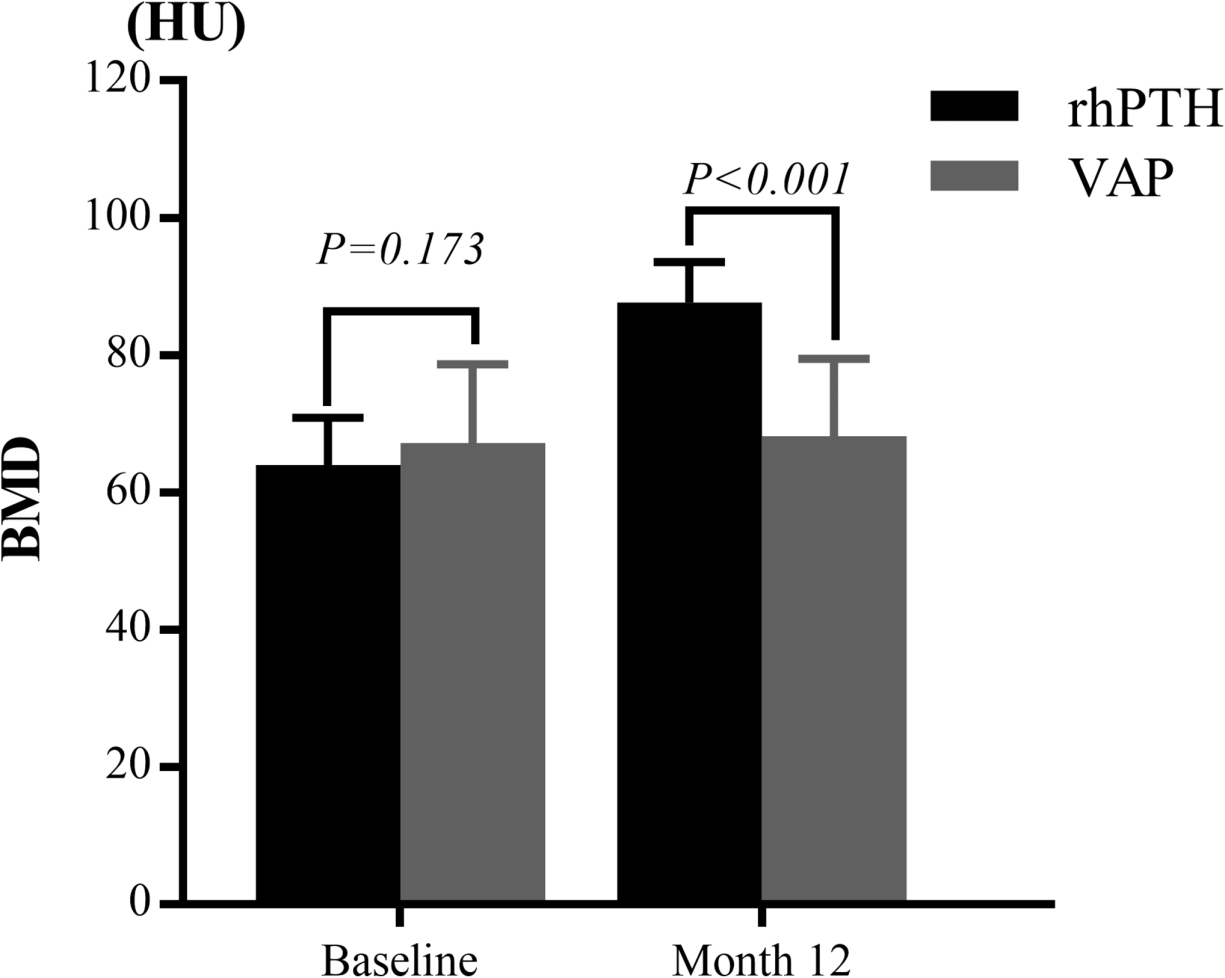
No significant differences in spinal BMD in the rhPTH and VAP groups at baseline (*p* = 0.173). At month 12, the BMD in the rhPTH group was significantly higher than in the VAP group (*p* < 0.001). BMD, bone mineral density

At baseline, no differences in spinal BMD assessed by L4 CT value were found between rhPTH (63.95 ± 6.93HU) and VAP groups (67.17 ± 11.55 HU) (*p* = 0.173). The value in the rhPTH group (87.66 ± 5.91HU) was significantly higher than the VAP group (68.15 ± 11.32HU) at month 12 (*p* < 0.001). The CT value of L4 increased markedly in the rhPTH group from baseline to month 12 (*p* < 0.001) but not in the VAP group (*p* = 0.212) (Fig. 6).

From baseline to month 12, significant differences were found in the BMD increment in the rhPTH (23.71 ± 6.12HU) and VAP groups (0.98 ± 5.43HU) (*p* < 0.001).

### Adverse Events

No serious adverse events occurred in either of the groups. No new OVCF occurred in the rhPTH group. However, eight patients experienced new OVCF following VAP. The new OVCF rate was higher in patients receiving VAP (*p* = 0.042).

### Sf‐36

No significant differences in the SF‐36, including eight subscale scores, MCS and PCS scores, were found between groups at baseline (all *p* > 0.05). At month 12, the SF‐36 in the rhPTH group was significantly higher than in the VAP group (all *p* < 0.05). (Table 3).

**TABLE 3:** Comparisons of summary and subscale scores of SF‐36 by Mann–Whitney *U* test

	rhPTH group	VAP group	Test statistic	*p*‐value
Summary scores				
Standardized PCS				
Baseline	17.04 ± 8.08	14.12 ± 8.22	0.033	0.116
Month 12	51.92 ± 2.41	44.78 ± 10.32	18.143	0.001
Standardized MCS				
Baseline	26.81 ± 5.75	27.52 ± 4.07	1.135	0.122
Month 12	61.85 ± 2.33	53.97 ± 11.30	31.796	0.002
Subscale scores				
PF (0–100)				
Baseline	30.91 **±** 19.00	24.90 ± 20.17	0.009	0.124
Month 12	93.18 ± 2.91	80.61 ± 17.76	23.675	0.001
RP (0–100)				
Baseline	3.41 **±** 8.78	2.55 **±** 7.65	0.679	0.675
Month 12	88.64 ± 12.74	67.86 ± 37.50	12.161	0.041
BP (0–100)				
Baseline	20.00 **±** 6.47	17.06 **±** 9.27	9.205	0.181
Month 12	88.36 ± 2.74	78.69 ± 20.16	9.989	0.017
GH (0–100)				
Baseline	42.14 **±** 8.81	41.22 **±** 9.92	0.744	0.752
Month 12	72.50 ± 10.77	63.43 ± 16.86	1.475	0.038
VT (0–100)				
Baseline	18.18 **±** 11.08	16.73 **±** 7.11	4.709	0.781
Month 12	89.32 ± 4.95	72.35 ± 21.53	19.171	0.001
SF (0–100)				
Baseline	20.20 ± 12.66	16.78 ± 11.37	2.674	0.463
Month 12	95.46 ± 5.59	79.37 ± 25.96	24.719	0.017
RE (0–100)				
Baseline	21.22 ± 31.79	27.21 ± 26.94	1.060	0.308
Month 12	100.00 ± 0.00	85.04 ± 22.63	53.575	0.001
MH (0–100)				
Baseline	28.55 **±** 12.55	27.27 **±** 10.79	2.319	0.739
Month 12	91.27 ± 3.63	77.55 ± 19.54	38.938	0.006

Abbeviations: BP, bodily pain; GH, general health; MCS, mental component summary; MH, mental health; PCS, physical component summary; PF, physical functioning; RE, role‐physical; RP, role physical; SF, social functioning; VT, vitality.

From baseline to month 12, all the increment of subscale scores, MCS and PCS scores in the rhPTH group were significantly higher than in the VAP group (all *p* < 0.05).

## Discussion

By the end of the follow‐up period in the present study, the VAS and ODI were better in the rhPTH group than in the VAP group. CT images confirmed the OVCF union in all patients in the rhPTH group. No significant differences in the changes in such imaging parameters as KA, ABH, and PBH were found between the two groups. The spinal BMD assessed by the CT value of L4 was higher in the rhPTH group than in the VAP group. The number of new OVCF was fewer in the rhPTH group than in the VAP group. The HRQoL assessed by the SF‐36 was higher in the rhPTH group than in the VAP. All the results collected confirmed that rhPTH was better than VAP in improving back pain and physical ability, promoting fracture union, increasing the spinal BMD to decrease the incidence of new OVCF and improving the HRQoL.

### Challenges of VAP Treatment

VAP can significantly and quickly reduce back pain by restoring the stability of the fractured vertebra. Recently, however, high‐ to moderate‐quality evidence indicated that VAP offered no clinical benefits compared with the sham procedure and did not recommend VAP to treat acute or subacute OVCF^16^, ^17^.

OVCF is the most severe complication of systemic osteoporosis, and the reduction of vertebral refracture risk should be one of the outcomes of OVCF treatment. Attention should be paid to treating both local fracture and systemic low bone mass. Hence, the OVCF and osteoporosis treatment to prevent new OVCF is vital in clinical practice. However, the bone cement injected into the fractured vertebra could not exert the effects either in boosting OVCF union or improving the whole spinal BMD. In contrast, the fracture risk increased significantly following VAP both in the cemented vertebra and adjacent vertebrae^18^, ^40^. Given the disadvantages of VAP, orthopaedic surgeons have been exploring effective treatments for acute OVCF.

### Advantage of rhPTH for OVCF

In our study, the VAS significantly decreased in the rhPTH group compared with the VAP group during the follow‐up. By the end of the follow‐up, the back pain and physical ability in the rhPTH group assessed by VAS and ODI were better than those in the VAP group.

The effects of rhPTH treatment on bone healing at various fracture sites have been described in the literature^41^, ^42^, ^43^. Different from previous studies, sagittal CT images were used to quantitatively assess the union of OVCF. Our study indicated that the bone bridge connecting the upper and lower endplates was detected on sagittal CT images of all the fractured vertebrae, confirming the enhancing effect of rhPTH on the bone union of OVCF.

Piazzolla *et al*.^44^ reported the bone union of OVCF related to the improved VAS and ODI. Unlike the mechanisms of VAP, rhPTH treatment gradually restored the continuity of fractured vertebrae. The gradually improved VAS and ODI showed that boosting OVCF healing following rhPTH treatment could exert more reliable clinical outcomes than VAP treatment during the entire follow‐up period.

There had been reports that VAP treatment could prevent progressive kyphosis and vertebral height loss^45^. At month 12, both KA and increment of KA were similar in both groups in our study. However, no changes in KA could fully demonstrate the loss of vertebral height following treatment. So, we further evaluated the changes in ABH and PBH of the OVCF vertebra. We found no statistical difference in the changes in ABH and PBH between groups at month 12. All measurements indicated that rhPTH treatment exerted a preventive effect on vertebral height loss.

Mikula *et al*.^46^ confirmed that rhPTH treatment could increase lumbar BMD assessed by routine CT. Our study's CT quantitative assessment of BMD was a better method than dual‐energy X‐ray absorptiometry^35^. The increased BMD of L4 was observed in the rhPTH group compared with no changes in the values in the VAP group.

According to Lindsay^47^, a new OVCF will occur again in approximately 20% of females within a year. The OVCF risk could be reduced by 40%–70% following rhPTH treatment^27^, ^48^. Compared with VAP, improved BMD, a surrogate determinant of increased vertebral strength^49^, may relate to lower OVCF risk in our study. By the end of the follow‐up, the rhPTH group had a lower rate of new OVCF than the VAP group.

OVCF was related to a negative impact on the HRQoL^50^. Chen *et al*.^51^ reported that rhPTH treatment showed better clinical outcomes with significantly improved HRQoL. Although SF‐36 improved by the end of follow‐up, the scores were higher in the rhPTH group than in the VAP group.

Compared with VAP, one advantage was rhPTH treatment with no hospital treatment required, reducing healthcare costs. Combining the improved clinical outcomes, bone union signs, ameliorated osteoporosis, and lower rates of new OVCF, rhPTH treatment could be an effective alternative to VAP for OVCF.

### Limitations

Our study showed the clinical outcomes of rhPTH for acute OVCF, especially in boosting OVCF union. However, multicentric randomized trials are necessary for future studies. Although vital for early treatment, we only focused on patients with acute OVCF in this study. Further studies are needed to assess the clinical efficacy of rhPTH on subacute and chronic OVCF. High‐density bone cement within the vertebral bodies affects the evaluation of bone bridge. We did not evaluate the fracture union of the affected vertebrae in the VAP group, which is another limitation. Additionally, the subsequent anti‐osteoporosis therapy with other anti‐osteoporotic medications should also be considered in the future.

### Conclusion

For acute OVCF patients, rhPTH could significantly increase bone mineral density to promote the radiographic union of the fractured vertebrae and reduce new OVCF. More importantly, it was superior to VAP in alleviating back pain and improving physical ability and health‐related quality of life, with no requirements for hospitalization.

## Author's Contribution

Pengguo Gou and Feng Chang conceived and designed the study. Pengguo Gou, Zhihui Zhao, and Chen Yu wrote the manuscript. Ting Zhang and Gang Gao performed the experiments. Xuefeng Hou performed the data analyses. All authors read and approved the final manuscript. Pengguo Gou and Feng Chang contributed equally to this manuscript. Pengguo Gou, Zhihui Zhao, and Chen Yu contributed equally to this manuscript.

## Conflicts of Interest

All the authors declare no conflict of interest.
